# Assessment of inhaled dust by workers and suspended dust for pollution control change and ergonomic intervention in metal casting industry: A cross-sectional study

**DOI:** 10.1016/j.heliyon.2020.e04067

**Published:** 2020-05-30

**Authors:** Wahyu Susihono, I Putu Gede Adiatmika

**Affiliations:** a Industrial Engineering Department, Faculty of Engineering, University of Sultan Ageng Tirtayasa, Banten, Indonesia; b Faculty of Medicine, University of Udayana, Bali, Indonesia

**Keywords:** Inhaled dust, Suspended dust, Ergonomic intervention, Metal casting industry, Engineering, Materials science, Environmental science, Health sciences

## Abstract

Metal casting industry including is an industry which produce high dust pollution (fly ash). Improvements in the form of ergonomic interventions have been carried out by many companies, but do not guarantee all parameters run well. The total indoor suspended dust (TSP) measurement results are not enough to guarantee healthy working conditions. Additional assessment of workers' inhaled dust is needed to change pollution control and work improvement to ergonomics. The design of this study is Cross Sectional Study. Research subjects numbered 84 people. All samples met the inclusion criteria. Measurement results of Characteristic of research subject, Working Environment Conditions, Exposition of dust inhaled by workers, Total Indoor Suspended Dust of the Company (p > 0.05). Found critical hours of workers exposed to dust (fly ash), starting from 4 h after working (Department of Process Cement, Department of Black Sand) and 2 h after working for the Department of Loam. Critical hours exposed to dust (fly ash) used as the basis for company management and regulators to take new policies in controlling fly ash pollution and ergonomic interventions. Ergonomic interventions can be carried out by activating the dust collector at critical hours, applying active resting hours at critical hours and conditioning workers to breathe fresh air. The impact of this ergonomic intervention is a decrease in musculoskeletal complaints by 25.27%, reduction in boredom 25.01%, and an increase in job satisfaction 38.46%.

## 1 Introduction

The metal casting industry in Indonesia can be found on the islands of Java, Sumatra and East Kalimantan. The metal casting industry generally takes the form of industrial centers and legacy industries so that the manufacturing process applied is not dynamic. A problem that has arisen for a long time and has not yet been resolved in the metal casting industry is air pollution in the form of dust [1, 2], which has an impact on reducing the health level of workers [3, 4, 5]. The government has tried to include dust control elements as parameters required in the issuance of operational licenses.

At this time, government policy in publishing business operational lisence only considers everyday Indoor Suspended Dust average of a company and has not considered inhaled dust by workers that its value might be higher. This condition should get more serious attention since regulation made to maintain workers' health and convenience in the industry. Inhaled dust is closely related to workers' physiological impact. Air quality is interpreted with air pollutant level. High inhaled dust can add workers' physiological burden. Air quality affects workers' health and satisfaction on the working place [6, 7, 8]. Good air quality has positive impact on workers' performance [9] and satisfaction [10, 11, 12]. Vice versa, air pollution in form of dust that goes beyond allowable threshold will affect workers' health either in short or long term.

Many companies have acquired their business operational permit after their everyday indoor suspended dust averages are known to be less than 10 mg/m^3^. However, it is found that company's indoor suspended dust average value is really fluctuating in each hour of measurement period per day. It is caused by working condition dynamics and type of job within the industry. Job in metal casting industry is included as extreme category. The workers are surely exposed to flying ashes especially in the industry which uses cupola or induction furnace. Metal casting industry is categorized as primary industry that potentially produces industrial waste in form of flying ashes. Generally the waste comes from imperfect combustion of various metals or even its supporting materials. The produced air pollution is the side effect of metal casting. Commonly the danger of metal processing industry is pollutant of quartz sand [13]. Quartz sand exposition to body physiological condition can increase lung inflammation and injury, leukocytes response, red blood cells, and production of macrophage [14]. Besides quartz sand, there is silica sand exposition. Additional production of metal casting is dust in form of silica. Silica sand can cause lung diseases [15, 16]. In another place, ashes from metal casting industry also contain nickel and arsenic [17].

Various improvement attempts have been applied, among others intervention of job improvement program [18]. The industry used as subject of this research has applied ergonomic concept in which human as the central of all improvements. Human becomes the main factor in job improvement. Nevertheless, in the improvement which has been performed (ergonomic intervention), there is something which still need to be more improved such as dust exposition to workers. The findings shows that the value of inhaled dust (fly ashes) by workers is higher than average of total indoor suspended dust (TSP). TSP value is found different in each working hours of the industry due to the complexity of working activity in the production floor. In general, activities of workers in metal casting industry are making cast, pouring molten steel into cast, and dismantling cast which the workers highly potential to be exposed of flying ashes in particular hours. Therefore, the management should measure and find out at what time the TSP value goes beyond threshold (10 mg/m^3^). Consequently, critical hours (maximal inhaled dust) for workers and then human-based improvement (ergonomic intervention) can be performed. Keywords of ergonomic intervention is capability or in accordance with users [19].

TSP value per-taking (hours) period also can be used as basic of changing national air pollution control policy by providing critical hours of dust beyond threshold. In consequences, the cost of air pollution control will be more efficient. Findings of this critical hour of maximum inhaled dust are also a part of warning system to the workers in metal casting industry. Hence, they will be discipline in working and use appropriate personal protective equipment. It is found that in the production floor working habit and culture in the industry of metal casting has not yet run effectively.

In this research, dust measurement will be applied in three departments namely department of Process Cement, Loam, and Black Sand. These three departments have their own differences in casting material mixing mechanism. Therefore, there are different chemical reaction that affects type and volume of dust including property material characteristic of dust. Eventually separate test needs to be performed.

The total value of the company's indoor suspended dust in hours can be used as a basis for ergonomic interventions that could be improving employee performance. Improved employee performance in this study in the form of decreased musculoskeletal complaints, decreased boredom, and increased job satisfaction. Industries with high dust pollution require special attention in handling worker health.

After knowing the results of the assessment of Inhaled Dust by Workers (units in days) and the total dust of the company suspended indoor measured in hours, then the quality of the company's performance can be determined. The average value of indoor suspended dust is used to determine the critical hours of workers exposed to dust. While the average value of workers' inhaled dust is used to determine the level of dust particulate exposure that is inhaled by workers every day. The measurement data is also used as a basis for improving ergonomic interventions. The application of ergonomics in a company needs to be evaluated periodically to improve the performance of employees and the company. The ergonomic concept also considers the use of cost-efficient, so this research can recommend at what time the dust collector should be turned on. This consideration can increase company profits through operational cost efficiency in the use of machines (dust collector).

## 2 Materials and methods

Inclusion criteria in this research are determined by several factors namely sample of this research. The sample is workers whose duties are doing metal casting process starting from preparation, pour molten steel to the cast, and dismantling cast. The workers age is around 20 years old until 40 years old, male, physically healthy proven by doctor statement letter, have normal BMI (between 18.5 until 25.0), at least have 4 years experiences, and last they are willing to be involved as sample until this research complete (proven by filling informed consent).

This research is taking place in metal casting industry which its products have obtained Indonesian National Standard (SNI) so that it becomes reference by other similar industries. The company produces nodular metal or flexible material property, physical sound that more aloud, and longer tensile strength compared to pig iron property material (hard, brittle). The company is committed to constantly operates during the research, there is no large scales improvisation that change recent work mechanism, and does not change working hour that could change working activity.

### 2.1 Sample collection technique

Target population is all employees in the metal casting industry (n = 210 workers). Accessible population is workers in the three departments production floor (n = 84 workers). Accessible population is determined by workers' name as the subject of the research, and workers' status as permanent employee in the metal casting industry.

Population that met inclusion criteria will be categorized into sampling frame. Age limitation is determined based on proportion of total population. Separation is performed due to the wide population age range so that sample distribution will be more even. The population is given registration number. The sample is taken by simple random sampling using random number table.

The company which is chosen as research place has induction kitchen and performed ergonomic intervention since 2015, so that working condition has been improved. As time passes by, evaluation and monitoring of dust condition must be performed since dust still becomes the problems that have not completely solved. Several referred and applicable ergonomic intervention until today is the use of ergonomic ladle (transportation means of molten steel with capacity of 50 kg), active break time for all workers to decrease working load, the use of display or dashboard as information media of duty and working target for metal casting, implementation, and working induction. Every morning subjects are directed by team chief and Production Manager. This ergonomic intervention has given improvement for employee performance physiologically. For example, the decreasing of musculoskeletal complaint, work fatigue, boring, employees' satisfaction, and skin surface temperature. However, dust from working activity in the industry which is still exposed to the workers needs more attention and ergonomic-based controlling attempt in the future. Until today, there is not yet the best strategy chosen. Consequently, monitoring and measurement of inhaled dust and total indoor suspended dust is needed. The data are collected since May 2018 until February 2019 (10 months). The data collection is performed in the first week for 4 days respectively and repeated once every 3 months.

### 2.2 Tools and procedure

Inhaled dust is the amount of dust (fly ash) inhaled by workers in breathing zone (around nose). It is measured by using *Portable Personal Air Sampler Pump* or it is called as *Personal Dust Sampler (PDS)* Brand of *F & J Speciality* made in USA with Gravimetry method. The measurement is conducted since the subjects start working until they finish (outside active and scheduled break time). The used tools are *Personal Dust Sampler (PDS)* or *Portable Personal Air Sampler Pump*, L-5P, Brand of F & J Speciality made in USA, Flow Range 0.86–6 LPM (800–6000 cc/min), with accuracy **±**5%, to measure dust inhaled by workers.

**Figure 1:**
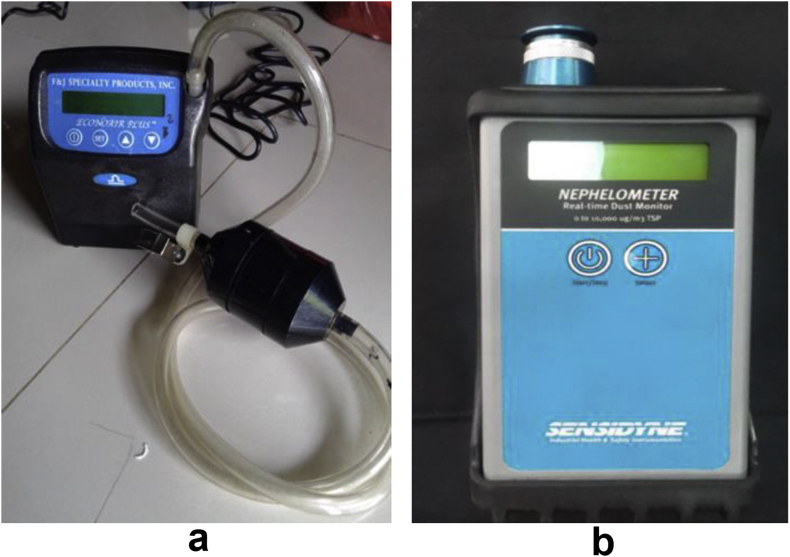
a. Personal Dust Sampler (PDS); b. Real-time Dust Monitor (RTDM).

Total of indoor suspended dust is fly ash particle which flies freely in indoor. Measurement is conducted by using *Nephelometer (Realtime Dust Monitor)*. The measurement is carried out in 9 indoor points. The measurement is performed gradually in which 2 h/day (1 day = 5 times measurement). The used tool is *Nephelometer (Realtime Dust Monitor)* brand of Sensidyne, with accuracy level of 0 until 10.000 μg/m^3^ *TSP*. Clearer Dust Sampler (PDS) and Real-time Dust Monitor (RTDM) can be seen in Figure 1.

The measurement is performed periodically in which every 3 months (first week for 4 times of measurement). Every worker is given PDS tool and must be used during the activity.

Indoor suspended dust measurement is performed every 1 h. Therefore, it cannot catch fly ash after the workers finish their work and PDS is needed to see dust inhaled by workers directly. Disassembling cast activity is the most extreme activity due to imperfect cooling from steel temperatures above 1200 °C to temperatures below 40 °C. This results in high volumes of dust that are inhaled directly by workers. Too dry casting material potentially turns into fly ashes and it has mass also time that freely fly in the air. At the time the dust is freely fly in the air, the higher potency for dust inhaled by workers within company environment.

The impact of implementing ergonomics interventions was measured using a questionnaire. Employee performance measures used in this study are the level of musculoskeletal complaints, the level of boredom and the level of satisfaction. Musculoskeletal complaints were measured by a Nordic body map questionnaire containing 28 items of information about parts of the body complained of pain [20]. Work boredom is measured by the boredom questionnaire [21]. While job satisfaction was measured by a questionnaire of 20 items Minnesota Satisfaction Questionnaire/MSQ) [22], all measurements were made after work (posttest).

### 2.3 Research protocol

To all the subjects of this research to the value of 84 people (consists of 28 workers in Process Cement department, 28 workers in Loam Department, and 28 workers in Black Sand department) purpose and goal of this research are explained. After they understand, all subjects are asked to fill and sign the informed consent.

Furthermore, physically diagnostic examination to all subjects is carried out by local Community Health Center doctor involving examination of height (cm) and weight (kg) by using *weighing and height measuring machine* brand of *Serenity* model ZT-120; blood pressure by using *Automatic Blood Pressure Monitor* model HEM-7111 brand Omron, and continued by writing down the subject's age, gender, BMI, and working experiences.

The measurement of first day in the first month is carried out with steps in form of: in the morning subjects are gathered in specific room for employee transit, sign presence, given brief explanation relating to the upcoming measurement by researcher that will be performed for 1 day. Subjects are gathered in the corner of metal casting station in which open space, directed by Team Chief and Manager Production that each worker must use the existed working method. Each subject is prohibited to do activities outside the arranged activities by the company. Subjects install PDS tool by attaching pump on the side belt, suction valve hose is pinned in the collar, and breathing tube is directed to around the nose. PDS is turned on by pressing ON button and then the subject starts to do working activities inside the production room. Subjects are not allowed to do activities outside what has been arranged. The activity is monitored by using hidden camera. OFF button is pressed right after the breaking time announced and PDS is worn again or in ON mode right after lunch or when starting work again. This activity is conducted by workers that become sample in Loam department, Black Sand department, and Process Cement department.

After the workers are doing activities for 30 min, environment condition is measured by measurement order starting from measuring indoor dust with *Nephelometer (Realtime Dust Monitor)* tool, the result is written down, and continued by respectively measure: air temperature, moisture, wind velocity, light intensity, and noise in 9 points of department area and then the result is recorded. Measurement of environment condition is repeated every 2 h (1 day = 5 times of measurement) for 8 days/period. This study was approved by the General Hospital Health Research Ethics Committee Dr. Moewardi, Faculty of Medicine-Sebelas Maret University, Number 165/II/HREC/2015.

### 2.4 Statistical analysis

Average and Deviation standard of all subjects of the research from three departments are measured. Subject characteristic data are analyzed based on age, weight, height, experience, sistolic, diastolic, and BMI. Afterwards, Data Normality is performed by using Kolmogorov-Smirnov test on the meaningful level of 5% (α = 0,05). Since normal data obtain all values (p > 0.05), thus different test of subject characteristic, working environment condition data, and inhaled dust by workers, also indoor suspended particles are performed through Anova test. Statistics analysis is carried out by using IBM SPSS 20.

## 3 Results

This section may be divided by subheadings. It should provide a concise and precise description of the experimental results, their interpretation as well as the experimental conclusions that can be drawn.

### 3.1 Characteristic of research subject

Data of research subject characteristic is needed to determine whether the three departments of subject research have any differences. When any differences are found, re-measurement needs to be done to keep the internal validity of research high. Several characteristics of subject that need to be measured can be seen in (Table 1). Age measurement based on date of birth from ID Card, gender is phenotypic characteristic shown by secondary gender traits, all samples are male. Weight measurement is determined by body weight measured with 0,2 kg accuracy level scale. Health level is a healthy status of the subject proven by statement letter from local Community Health Center doctor (Measurement of sistolic, distolic, BMI). Complete result of measurement in (Table 1).

**Table 1:** Subject characteristic data.

Description	Department of Process Cement (N = 28)	Department of Loam (N = 28)	Department of Black Sand (N = 28)	*p*-Values
Age (year)	36.5 ± 3.2	35.6 ± 3.5	35.3 ± 3.9	0.77
Weight (kg)	60.8 ± 2.6	62.4 ± 2.2	61.8 ± 2.2	0.28
Height (cm)	163 ± 3.2	163 ± 2.9	163.4 ± 3.1	0.65
Working Experience (year)	17.57 ± 6.8	19 ± 6.6	18.64 ± 7.1	0.27
Sistolic (mmHg)	118.5 ± 3.5	119.8 ± 1.5	120 ± 1.4	0.57
Diastolic (mmHg)	75.9 ± 4.7	77.9 ± 4.8	77.6 ± 1.4	0.37
Body Mass Index (kg/m^2^)	22.9 ± 1.2	23.5 ± 0.9	23.2 ± 0.8	0.07

Research subject is in total of 84 people (consists of 28 people in Cement Process department, 28 people in Loam department, 28 people in Black Sand department), all of them are male with average age in total 35.80 ± 3.4 years (Table 1). It means that the workers have acclimated by working environment condition. During research there are no subjects that decide to drop out, so it means all data can be processed and analyzed. All subject characteristic data shows value of (p > 0.05) which means subject characteristic data are not having any significant differences among three departments. Workers in three departments have same characteristics such as age, weight, height, and working experience in health condition of sistolic and diastolic as well as BMI (Table 1).

### 3.2 Working environment condition

Every sample collection needs data of physical environment condition. The data are used as foundation in keeping internal validity. Company's environment condition data have no differences (p > 0.05) so that company's environment in each measurement is the same. In general, change of wind velocity, humidity, and air temperature affect the assessment level of each variable examined (dust inhaled by workers and total indoor suspended dust). Therefore, there is consistency of working environment data on each data collection period. Since the three departments are in one workshop, separated with 1,5 m separator, so that measurement point determination is carried out thoroughly. Production floor area (P20/L11 = 220 m). Determination of sample collection point symmetrically interval of 3m P/L, except edge of 2.5m, therefore 40 points are obtained.

Every sample collection of inhaled dust and total indoor suspended dust needs measured physical working environment data that are involving air temperature, humidity, wind velocity, light intensity, and noise. All data of company working environment condition show the value of (p > 0.05), it means company's working environment condition data are not significantly different in each data collection (Table 2). The highest air temperature is on the 4th month namely 29.5 ± 1.3 °C, relative humidity on the 4th month namely 78.6 ± 7.2%, light intensity on the 4th month namely 132.1 ± 23.87 Lux, noise intensity on the 1st month namely 67.5 ± 0.9 dBA, and constant wind velocity.

**Table 2:** Data of Company's working environment condition.

Variable	1^st^ Month (N = 40 points)	4^st^ Month (N = 40 points)	7^st^ Month (N = 40 points)	10^st^ Month (N = 40 points)	*p*-Values
Air temperature (^0^C)	28.5 ± 1.2	29.5 ± 1.3	28.6 ± 2	29.1 ± 1.1	0.07
Relative Humidity (%)	79.6 ± 4.9	78.6 ± 7.2	77.1 ± 6.5	77.5 ± 7	0.07
Lighting Intensity (Lux)	131.2 ± 47.6	132.1 ± 23.87	131.2 ± 50.7	131.6 ± 34.3	0.92
Noise Intensity (dBA)	67.5 ± 0.9	67.1 ± 1.2	67.07 ± 1.7	67.2 ± 2.5	0.78
Wind Velocity (^m^/_det_)	0.7 ± 0.01	0.7 ± 0.01	0.7 ± 0.01	0.7 ± 0.01	0.65

Information and company working environment data are needed before data collection of inhaled dust and total indoor suspended dust since if there any working environment differences, it will affect different treatment condition as well. Dust condition is in free air will also be different. Hence, data collection of working environment condition need to be maintained to not bring any differences. If differences appear, data collection must be repeated to keep the internal validity. Working environment condition greatly influences characteristic and attribute of fly ashes in the air such as air temperature, relative humidity, and wind velocity.

### 3.3 Exposition of dust inhaled by workers

After environment condition is measured and there is no significant difference, dust inhaled by workers in three departments is measured. The result of measurement of inhaled dust by workers in three departments by using PDS in its each period can be seen in (Table 3).

**Table 3:** Measurement result data of inhaled dust by workers in three departments by using PDS.

Variable	1^st^ Month (N = 28 worker)	4^st^ Month (N = 28 worker)	7^st^ Month (N = 28 worker)	10^st^ Month (N = 28 worker)	*p*-Values
Dust inhaled by workers in Process Cement department (mg/m^3^)	33.8 ± 6.4	32.9 ± 5.9	29.5 ± 8.5	31.3 ± 2.1	0.25
Dust inhaled by workers in Loam department (mg/m^3^)	40.5 ± 7.1	39.4 ± 4.0	41.2 ± 2.2	42.2 ± 6.9	0.43
Dust inhaled by workers in Black Sand department (mg/m^3^)	48.5 ± 4.8	56.3 ± 7.9	50.6 ± 1.6	50.7 ± 3,2	0.57

Based on (Table 3), it shows that the highest value of dust inhaled by workers in Black Sand department with average of 51.52 mg/m^3^ during 10 months research period. Meanwhile, dust inhaled by workers in Loam department is in average of 40.83 mg/m^3^, and the smallest value of dust inhaled by workers is in Process Cement department with average of 31.63 mg/m^3^. The three departments have variation of dust inhaled by workers average and in a whole all of the values are beyond the allowable threshold (<10 mg/m^3^).

All data of dust inhaled in three departments have value of (p > 0.05), it means that data collection from the 1st, 4th, 7th and 10th month are not significantly different. Consistency of this data shows that there is no management or work mechanism change during the research period. Dust inhaled by workers on each data collection is not significantly difference (Table 3). This research's weakness is dust inhaled by workers values that are presented in cumulative form, and by looking at fluctuation of standard deviation data it can be predicted that dust exposition level is fluctuated in each worker. Dust exposition data in each worker are not presented since the limitation and confidentiality of individual data in the company.

On each worker the difference of dust exposition acceptance level is found, so that impact on the body will also be different. Based on (Table 3), it can be known that there are differences of dust volume caught by filter after the workers are active in one business day.

**Figure 2:**
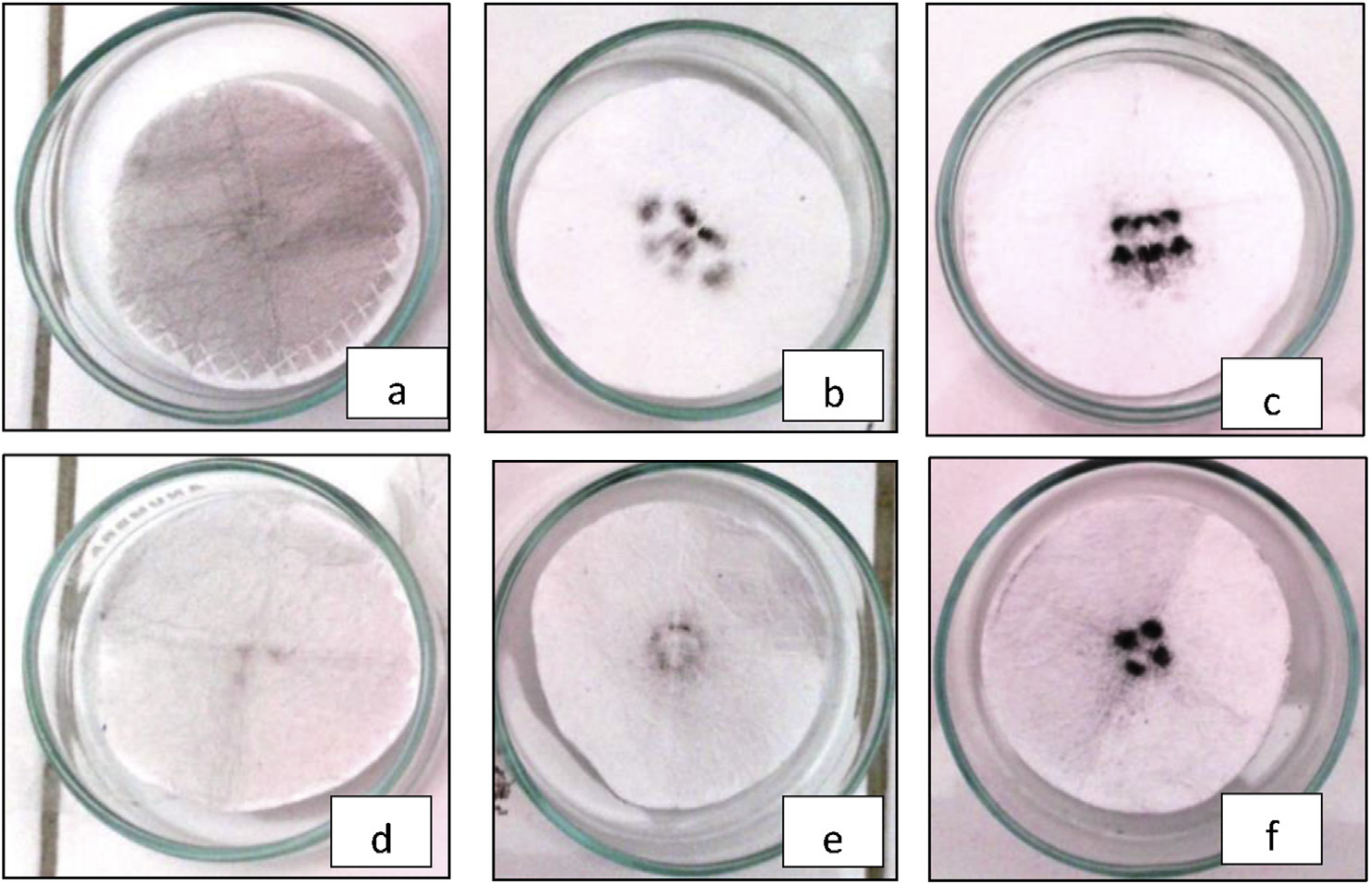
Dust inhaled by workers from: a. Department of Process Cement (The most caught fly ashes volume); b. Department of Loam (The most caught fly ashes volume); c. Department of Black Sand (The most caught fly ashes volume); d. Department of Process Cement (The least caught fly ashes volume); e. Department of Loam (The least caught fly ashes volume), f. Department of Black Sand (The least caught fly ashes volume).

Based on (Figure 2a,d), caught fly ashes volume in Department of Process Cement compared to (Figure 2b,e) caught fly ashes volume Department of Loam have different characteristics and forms. Fly ashes in Process Cement department tend to spread or even in filter while fly ashes from Loam department tend to in even round form. Meanwhile, in (Figure 2c,d), it is found that fly ashes in Black Sand department are in form of black-round and centered in particular point. If it is compared to volume in (Table 3), fly ashes with black-round form and centered in particular point has the highest volume compared to other forms.

### 3.4 Total indoor suspended dust of the company

Total of indoor suspended dust is measured by using Nephelometer *(Realtime Dust Monitor),* and observation in nine points of three departments is carried out. Measurement is done 5 times per day or total indoor suspended dust is measured with 2 h interval except the initial measurement that is performed 30 min after starting.

Based on (Table 4), exposition of total indoor suspended dust is extremely dangerous for workers since in the particular hours it goes beyond allowable threshold value (NAB). Maximal value of fly ashes exposition to the workers is 10 (mg/m^3^). In the industry of metal casting, occurrence time of particle exposition varies greatly in each department which is indicated by total suspended particle (TSP).

**Table 4:** Measurement result data of total indoor suspended dust particle in three departments by using RTDM.

Measurement Time	TSP, 1^st^ Month (mg/m^3^), (N = 9 points, 4 days)	TSP, 4^st^ Month (mg/m^3^), (N = 9 points, 4 days)	TSP, 7^st^ Month (mg/m^3^), (N = 9 points, 4 days)	TSP, 10^st^ Month (mg/m^3^), (N = 9 points, 4 days)	Inf.	*p*-Values
**Department of Process Cement**						

30 min after working	1.066 ± 0.1	1.511 ± 0.3	1.409 ± 0.4	1.081 ± 0.1	< NAB	0.99
2 h after working	9.406 ± 2.5	9.441 ± 2.4	8.217 ± 3.4	8.527 ± 3.5	< NAB	0.95
4 h after working	13.959 ± 2.3	12.102 ± 2.1	13.048 ± 2.2	12.765 ± 2.4	> NAB	0.99
6 h after working	14.073 ± 1.3	9.239 ± 2.6	10.406 ± 1.4	9.328 ± 2.2	> NAB	0.91
6 h after working	0.939 ± 0.3	0.871 ± 1.0	1.895 ± 0.7	1.494 ± 0.4	< NAB	0.61
Average per day	7.889 ± 5.5	7.433 ± 4.9	6.995 ± 4.3	6.639 ± 4.3	< NAB	0.60

**Department of Loam**						

30 min after working	0.567 ± 1.0	0.932 ± 0.5	1.817 ± 0.3	0.608 ± 0.5	< NAB	0.11
2 h after working	5.828 ± 2.7	9.097 ± 3.1	12.143 ± 2.4	11.725 ± 2.6	> NAB	0.56
4 h after working	9.477 ± 2.4	10.201 ± 1.6	10.400 ± 1.7	10.540 ± 3.1	> NAB	0.12
6 h after working	10.716 ± 3.9	9.775 ± 2.4	9.775 ± 3.2	8.050 ± 3.1	> NAB	0.79
6 h after working	1.902 ± 0.4	1.049 ± 0.3	1.555 ± 0.5	1.287 ± 0.6	< NAB	0.08
Average per day	5.698 ± 3.6	6.211 ± 4.2	7.138 ± 4.4	6.442 ± 4.4	< NAB	0.56

**Department of Black Sand**						

30 min after working	0.860 ± 0.4	0.776 ± 0.5	1.293 ± 0.5	0.736 ± 0.7	< NAB	0.95
2 h after working	4.490 ± 3.3	6.097 ± 2.1	8.620 ± 1.2	7.027 ± 1.1	< NAB	0.36
4 h after working	9.327 ± 3.2	10.029 ± 2.5	10.327 ± 2.2	11.595 ± 2.2	> NAB	0.62
6 h after working	8.891 ± 2.1	8.295 ± 1.5	9.871 ± 2.6	9.322 ± 1.6	< NAB	0.22
6 h after working	0.980 ± 0.8	1.231 ± 0.5	2.231 ± 0.4	0.902 ± 0.3	< NAB	0.54
Average per day	4.910 ± 3.4	5.286 ± 3.4	6.468 ± 3.8	5.916 ± 4.1	< NAB	0.98

**NAB:** Threshold Value; **TSP:** Total Suspended Particle.

Result of measurement (Table 4) shows that in Process Cement Department, critical hours with particle exposition beyond NAB starts from the 4th hour until 6th hour after started working (at 11.00 until 13.00 WIB), while in Loam Department it is started from the 2 nd h until the 6th hour after started working (at 09.00 until 13.00 WIB), and in Black Sand department it is started from the 4th hour until 5th hour after started working (at 11.00 until 12.00 WIB). Collection of total indoor suspended dust in each measurement hour varies greatly. The variation really determines mechanism of fly ashes pollution control within respective department.

Result of critical hours becomes attention for management in determining pollution control. Differences of the fly ashes exposition critical hours give recommendation to do intervention that side with workers and control pollution within the industry. Averages of total indoor suspended dust in the three departments have no significant difference (p < 0,05). The value shows that the each three months measurement for 10 months shows the same value.

## 4 Discussion

Variable of subject characteristic and environment condition in each measurement period obtain the same value (p > 0.05). This condition indicates that the company does not perform any changes of working system and layout. Working environment consistency is used to keep data internal validity. Changes on inhaled dust and total indoor suspended dust are influenced by the workers condition itself not from working environment.

Average respondents' age is 35.80 ± 3.4 years old and it indicates an appropriate age to work. This age is included as productive age. Moreover, BMI range around 23.2 ± 0.8 kg/m^2^, which means the workers are in normal condition. Sistolic and diastolic values is in the normal condition and fit to go working. Experience level with average 18.64 ± 7.1 shows that the workers are acclimatized and accustomed to work regularly in the metal casting activity.

In general, change of wind velocity, humidity, and air temperature affects assessment level of respective examined variable. Thus, working environment data consistency in each data collection period is needed. Since the three departments in one workshop separated by 1,5m tall separator, the determination of measurement point is carried out thoroughly. Floor production are (P20/L11 = 220 m). Determination of sample collection point symmetrically with interval 3m P/L, except edge 2.5m, so that it obtains 40 point.

Working environment highly affects research variables. Sample collection with different working environment will be indicator of working method or system change applied in the industry. The same production floor condition from the first period or until the last period of measurement is a form of working completion consistency. Therefore, inhaled dust and total indoor suspended dust data can be seen and compared in each research period. Moreover, company's working environment in each period shows consistent value. The air temperature of 28 °C is the threshold of convenience for working category. Temperature is defined as physical unit that states environment or body's hot or cold level. Workers performances tend to decrease when they reach temperature above 25 °C [23]. Temperature increase also affects workers aggressive behavior [24]. Hence, the purpose of this convenience temperature is to optimize workers' performance [25, 26, 27]. In this research, relative humidity measurement result is 77% and it is included as convenience to work in Indonesia's company working environment. Humidity has an effect on workers physiological parameter and workers performance [28].

Light intensity for about 132.1 ± 23.87 Lux. This light intensity need to be increased to make working room brighter. Appropriate light intensity gives convenience to workers [28], also increases health and workers performance [29]. The purpose of appropriate light intensity will eliminate working mistake and accident [30]. Light adjustment needs to be done immediately so that health and performance of workers will increase [31].

Noise intensity in this research is 67.07 ± 1.7 dBA. The value shows workers can work for 8 h (<85dBA). If noise value is beyond the threshold, it will give negative effect on human health and performance [32] as well as other effects [33, 34, 35]. Noise is defined as an unwanted voice [36]. If it is further examined, noise and light intensity cause psychological symptoms of workers [37]. Moreover, result of wind velocity measurement in this research is constantly in the value of 0.7 ± 0.01 ^m^/_det_, and the value shows there is no air circulation change around the working environment.

### 4.1 Dust inhaled by workers

Air pollution is deemed to put human health in danger. Environment pollutant cause various disease such as cardiopulmonary and lungs cancer [38, 39]. Particle size and chemical composition of the dust particle itself give alteration on body biological response [40, 41]. Several researches are using animal model to prove nanoparticle causes inflammation response when it is inhaled or injected as well as it causes chronic inflammation disease such as fibrosis and cancer [42, 43]. Furthermore, it is found that exposition value of everyday dust inhaled by workers is beyond the threshold (>10 mg/m^3^). This condition obviously harms workers. Based on (Table 3), the highest value of dust inhaled by workers is in Department of Black Sand to the value of 51.52 mg/m^3^, Department of Loam 40.83 mg/m^3^, and Department Cement Process with average of 31.63 mg/m^3^. These three conditions are beyond allowable threshold by government (<10 mg/m^3^). Hence, control over dust exposition is immediately needed.

This research states that dust size highly affects body [44]. Bigger particle size is gathered in nasopharynx area (5–30 mm), but smaller particle size (1–5 mm) is gathered in tracheobronchial area. Smaller particle (<1 mm) and nanoparticle (<100nm) can penetrate to alveolar area. Moreover, particle size under 50 nm can directly interacted with lung epithelium. Long term effect wil arise for workers who inhale dust [45]. The use of personal protective equipment needs to be evaluated so that precise APD will be selected to maintain workers health. Even though physically workers condition can work and finish their determined target, but slowly their life quality will constantly decrease. Inhaled dust can cause allergy [46, 47, 48]. Other researches on metal casting industry have reported that particle size and type constituted dust bring danger to workers physiological condition [49].

Dust which is entered into respiratory tract can cause non-specific defense reaction since smooth muscles around the respiratory tract are stimulated and lead to narrowing. According to Deviandhoko [50], dust accumulation in lungs happen when we are breathing and inhaled air. The track passed by dust is nose, pharynx, trachea, bronchi, bronchioli and alveoli. Dust particles with size 0,3 μ follow brown movement while dust particles with bigger size of 0,6μ are restrained in the upper respiratory tract [51]. If dust enters alveoli, it will make alveoli tissue harden (*fibrosis*). If 10% alveoli suffers from fibrosis, the elasticity to contain air volume will decrease, the ability to bind oxygen and lungs capacity will decrease as well. Furthermore, this research only measures two variables. Thus, for further research it is recommended to measure workers physiological condition in order to discover lungs health. Other researches reported that workers exposed by dust are not always suffering from lungs capacity decrease due to their immune system, breathing strength, and ability to filter dust [50]. Nevertheless, working environment of metal casting significantly influences blood protein concentration [52].

Change of inhaled dust level on each department affected by material characteristics and process occurred, including measurement time condition [53]. Dust characteristic in Black Sand department is the raw material used continuously in recycle, additional materials in form of water to make steel cast, thus dryness level of the materials is incredibly high. This condition then potentially emerges high volume of fly ashes. In Process Cement department, cement is always added in each period of steel cast and fly ashes created from this mixing composition change. Meanwhile, in Department of Loam, cast making materials have the most weight so that its materials properties settles quickly. Nevertheless, the produced gas is quite high so that working behavior greatly influences high inhaled dust.

Up until now, data of dust inhaled by workers have not yet become requirement to determine company's performance. Findings of this research show that value of dust inhaled by workers in metal casting is really high. Hence, inhaled dust by workers parameter needs to be added to determine company's performance.

### 4.2 Total indoor suspended dust

Research relating to suspended particle has been conducted in several other working conditions [54, 55, 56]. Other researches reported that metal casting environment is highly dangerous due to dangerous chemical emergence simultaneously [57]. Particle size, amount, and exposition duration in working area are related to working health [58]. Dust in form of nanoparticle influences body function and immune response of workers in metal casting industry [59]. Based on Circular Letter Ministry of Manpower No.01/SE/MEN/1997, Ministry of Manpower and Transmigration No 13/MEN/X/2011, SNI 19-0232-2005, threshold (NAB) of metal dust stipulated in Indonesia is maximal to the value of 10 mg/m^3^. Result of total indoor suspended dust is averagely under the allowable threshold (<10 mg/m^3^). However, if we pay more attention to each working hours period there will be found various different values. In particular hour, workers exposed by dust or in other words dust level reaches (>10 mg/m^3^). In Process Cement Department, critical hours of particle exposition above NAB is starting from the 4th hour after started working until the 6th hour (at 11.00 until 13.00 WIB), while in Department of Loam it is started from the 2 nd h to the 6th hour after started working (at 09.00 until 13.00 WIB), and Department of Black Sand from the 4th hour until the 5th hour after started working (at 11.00 until 12.00 WIB). Therefore, this finding of fly ashes exposition critical hours becomes the concern for management and policy maker.

Findings of dust exposition critical hour are influenced by measurement result of working environment condition such as air temperature, humidity, and light intensity that are different in each country or industry location. Several countries with high humidity will be different if it is compared to countries with cold air temperature condition. It is also applicable for dust exposition level. If measurement is not performed in detail, it will cause inaccurate health improvement recommendation due to dust exposition [60].

Findings of this fly ashes exposition becomes reference and must be attention to management and policy maker to control fly ashes pollution. New policy should side with workers and one of the used policies is ergonomic intervention. Pollution control policy in metal casting industry should be distinguished with policy of other industries in general. At the critical hours, workers of metal casting industry are obligated to use personal protective equipment with strict supervision or if it is needed several ergonomic improvements need to be planned to decrease indoor suspended dust. Among others the attempts are dust collector tool addition and air circulation system repair [61]. Nonetheless, these tools need relatively expensive investment so that appropriate design technology based on user needs must be reviewed. Based on findings of dust inhaled by workers and dust exposition critical hours, government should perform evaluation in determining an industry performance quality. Findings of this research can be used as new reference to change pollution control mechanism. Human-based improvement (ergonomic intervention) is necessary to be re-evaluated.

Implementation of ergonomic concept decreases injury risk, increases productivity and work quality [62]. Ergonomic intervention is a participative mechanism to improve working areas and facilities [63]. Further measurement of working environment should consider macro ergonomic so that working quality in a company can be depicted [64].

Raw materials as the source of pollution source (fly ashes), if it is possible, must be chosen that minimally produce dust. Nevertheless, up until now it is hard to be performed. In addition, change working method by using supporting tool of ergonomic to make the workers is indirectly exposed to dust. Hence again, it is also hard to be performed since workers have been worked for 18 years and included as community technology.

The strength of this research is data collection on controlled environmental conditions, that is data collection can only be done when the company's working environment is the same (p > 0.05). Given the weather very determines the internal validity of research results. In addition, the consistency of work mechanisms including the number of workers and the commitment of workers and companies during the study period makes the research data valid and can be further processed. Improvement of pollution control can be done by adding parameters in the form of data on the average value of company suspended suspended dust (units in hours) and the average value of workers inhaled dust (units in days). The average value of indoor suspended dust (unit hours) is used to determine the critical hours workers are exposed to dust. While the average value of workers' inhaled dust (unit of day) is used to determine the amount of workers exposed to particulate dust. Another control is to carry out ergonomic interventions, namely by implementing resting hours after working 4 h and activating dust collector at critical hours.

Weakness of this research is examination of workers lungs condition that has not yet performed. Therefore, it is have to be perfected in the further research. In addition, further research also needs to establish the relationship between workers physiological condition and workers convenience level since averagely workers experience are beyond 18 years. By seeing this working experience, it can be interpreted that workers have endured longer and been accustomed to high dust exposition in metal casting industry. Besides macro ergonomic intervention, the company should evaluate working condition thoroughly [65]. Several similar researches in Pakistan with the same subject give recommendation to improve working condition [66]. In addition, further research also needs to establish the relationship between workers physiological condition and workers health level caused by fly ashes. Work improvement considers working condition since working environment really influences intervention adoption. New working habit needs time and strategy by considering human factor in each work improvement.

### 4.3 Impact of ergonomic interventions

In an industry that has implemented technology that is completely automated, workers are still found to experience musculoskeletal complaints [68], so that ergonomic intervention is needed as a work improvement. Ergonomic interventions can reduce complaints of posture [69]. Musculoskeletal complaints are caused by workplace design [70], and excessive physical activity [71].

Data was collected through filling out musculoskeletal complaints, boredom and job satisfaction questionnaires by study subjects in the 1 st month and 10 th month of the study. Data changes are calculated and statistically tested. Data on changes in employee performance after ergonomic interventions are presented in Table 5.

**Table 5:** Data on changes in worker performance after ergonomic interventions (n = 84).

Variable	Condition		Change (%)	Value	*p*-values
	1^st^ month	10^th^ month			
Musculoskeletal complaints	87.45 ± 1.53	65.35 ± 0.95	25.27^(a)^	31.31^t^	0.000
Boredom	86.45 ± 3.71	64.83 ± 1.84	25.01^(a)^	-3.31^z^	0.001
Satisfaction	42.82 ± 2.33	59.29 ± 1.11	38.46^(b)^	-11.93^z^	0.000

Note: (a) improvement; (b) decrease; t = different test with parametric analysis; z = different test with non-parametric analysis.

Based on Table 5 it is known that the impact of ergonomic interventions on workers is a decrease in musculoskeletal complaints by 25.27%, decrease in boredom 25.01%, and an increase in satisfaction 38.46%. Ergonomic interventions carried out were the use of ergonomic ladles, the provision of active rest periods, the use of displays, and the application of work induction that was applied during 10 months of work. Decreased musculoskeletal complaints generally occur in the neck, knees, back, legs, wrists and arms. This is in line with previous research, namely that generally musculoskeletal complaints occur in several parts of the body, including the neck, shoulders and knees [72], neck, back [m6], legs, upper arms, wrists and arms [73]. In general, work posture improvement can reduce musculoskeletal complaints in the head, neck, arms [74]. During the ten months, ergonomic intervention not only changes the level of musculoskeletal complaints, but also the level of boredom and satisfaction of employees.

If working conditions are comfortable, musculoskeletal complaints will go down at a certain time [75]. Changes in musculoskeletal complaints are experienced after 1 year of intervention [76], or after six 6 months of intervention [77]. The difference is influenced by the mechanism and type of intervention to workers. Ergonomic interventions also lead to increased satisfaction and decreased boredom. Ergonomic interventions in the form of providing active rest time create pleasant conditions so that workers feel satisfied with the results of their work. Ladle design interventions by accommodating workers' ideas can increase satisfaction. Remembering satisfaction is an individual's feelings and reactions to the work environment [78].

The results of this study perfect the ergonomic interventions that have been carried out previously, those are by providing resting hours after working 4 h, conditioning workers to breathe fresh air, and activating dust collectors at critical hours. These things are believed to improve employee health. The end result of an ergonomic intervention is an improvement in the quality of life of workers. The quality of life of workers can be used as material for further research in the future.

## 5 Conclusions

Every dust inhaled by workers is found to excess threshold value (>10 mg/m^3^). This condition really harms workers. Result of total indoor suspended dust averagely under the allowable threshold stipulated by government regulation (<10 mg/m^3^), but if it is examined clearly, measurement in each working hour finds different level of dust. There are critical hours of dust exposition to the workers.

In Process Cement Department, critical hours of dust exposition is (at 11.00 until 13.00 WIB), in Loam Department (at 09.00 until 13.00 WIB), while in Black Sand Department (at 11.00 until 12.00 WIB). These findings can be foundation for company management and government in making new policy to control fly ashes pollution and ergonomic intervention, which has been performed until now. New policy must take side with workers as in concept of ergonomic science. Policy should take side with workers health in the industry. Moreover, policies of pollution control in metal casting industry have to be distinguished with other industries policy in general.

Other controls are through ergonomics interventions, those are by applying rest hours after working 4 h, conditioning workers to breathe fresh air, and activating dust collectors at critical hours. Ergonomic interventions reduce musculoskeletal complaints by 25.27%, reduce boredom by 25.01%, and increase job satisfaction by 38.46%.

The recommendation of this study is that the Indonesian government needs to add data parameters on the average value of company suspended dust (units in hours) and the average value of workers' inhaled dust (units in days) as a requirement to issue operational permits. The average value of indoor suspended dust (unit hours) is used to determine the critical hours workers are exposed to dust. Whereas the average value of workers' inhaled dust (units of days) is used to find out the amount of workers exposed to particulate dust. Any improvement in working environment conditions should always be centered on humans.

## Declarations

### Author contribution statement

Wahyu Susihono: Conceived and designed the experiments, Performed the experiment, Wrote the paper.

I Putu Gede Adiatmika: Analyzed and interpreted the data, Contributed reagents, materials, analysis tools or data, Wrote the paper.

### Funding statement

This work was supported by the Lecturer Reflection Activities in 2019, the Program of the Directorate of Career and HR Competence, Directorate General of the 10.13039/501100009509Ministry of Research, Technology and Higher Education; University of Sultan Ageng Tirtayasa: Partnership the Postgraduate Program of Udayana University, according to the contract Number T/155/D2.3/KK.04.03/2019, September 27, 2019.

### Competing interest statement

The authors declare no conflict of interest.

### Additional information

No additional information is available for this paper.
